# Increased concentration of two different advanced glycation end-products detected by enzyme immunoassays with new monoclonal antibodies in sera of patients with rheumatoid arthritis

**DOI:** 10.1186/1471-2474-11-83

**Published:** 2010-05-03

**Authors:** Richard Vytášek, Liliana Šedová, Vladimír Vilím

**Affiliations:** 1 Department of Medical Chemistry and Biochemistry, 2nd Faculty of Medicine, Charles University, V Úvalu 84, Praha, CZ-150 06, Czech Rep; 2 Institute of Rheumatology, Na Slupi 4, Praha, CZ-128 50, Czech Rep

## Abstract

**Background:**

Levels of pentosidine (representative of advanced glycation end-products) in sera of patients with rheumatoid arthritis are increased when compared with sera of other diagnoses or healthy controls. These levels have been reported to correlate with clinical indices of rheumatoid arthritis activity and with laboratory markers of inflammation. The purpose of this study was to find out if these findings pertain to other advanced glycation end-products.

**Methods:**

We have developed two immunoassays based on new monoclonal antibodies to advanced glycation end-products. Antibody 103-E3 reacts with an unidentified antigen, formed in the reaction of proteins with ribose, while antibody 8-C1 responds to N^ε^-(carboxyethyl)lysine. We have used these monoclonal antibodies to measure levels of advanced glycation end-products in sera of patients with rheumatoid arthritis, systemic lupus erythematosus, osteoarthritis, and healthy controls. We calculated the correlations between advanced glycation end-product levels in rheumatoid arthritis sera and the Disease Activity Score 28 (DAS28), age, disease duration, CRP, anti-CCP, rheumatoid factor and treatment with corticosteroids, respectively.

**Results:**

Levels of both glycation products were significantly higher in sera of patients with rheumatoid arthritis when compared with sera of patients with systemic lupus erythematosus, osteoarthritis, or the healthy controls. Neither the level of N^ε^-(carboxyethyl)lysine nor the level of the 103-E3 antigen in rheumatoid arthritis sera correlated with the DAS28-scored rheumatoid arthritis activity. The levels of both antigens in rheumatoid arthritis sera did not correlate with age, gender, corticosteroid treatment, or levels of CRP, anti-CCP antibodies, and rheumatoid factor in sera.

**Conclusions:**

We report highly specific increases in the levels of two advanced glycation end-products in sera of patients with rheumatoid arthritis. This increase could be explained neither by rheumatoid arthritis activity nor by inflammation. We propose a working hypothesis that presumes the existence of a link between advanced glycation end-product formation and induction of autoimmunity.

## Background

Nonenzymatic glycation of proteins consists of a series of reactions between reducing carbohydrates and amino groups of proteins. Labile Shiff bases that are formed in the first step rearrange reversibly to the more stable Amadori products. A plethora of permanent compounds, formed from Amadori products in complex reaction pathways, have been designated as advanced glycation end-products (AGE). The currently-known AGE can be classified into three major groups: non-cross-linking species (e.g., N^ε^-(carboxymethyl)lysine, CML) [1], fluorescent cross-links (e.g., pentosidine) [2], and non-fluorescent cross-linking species (e.g., glucosepan). Many excellent reviews have described the reaction pathways of AGE formation, their structure, relationship to aging and disease, and other facets of the field [3].

Thus far, all studies aimed at measuring AGE levels in serum or plasma of patients with rheumatoid arthritis (RA) have been limited to exclusively measuring pentosidine using high-performance liquid chromatography (HPLC) [4-12]. RA sera were compared with sera of healthy controls [4,7,8], with sera of patients with osteoarthritis (OA) [6], with sera of patients with systemic lupus erythematosus (SLE), or with sera of patients with diabetes [5]. Pentosidine levels in plasma of RA patients were compared with levels in plasma of patients with OA, with levels in plasma of patients with diabetes, and with levels in plasma of normal subjects [12]. Each of these studies reported an increased mean level of pentosidine in RA serum/plasma. Furthermore, Takahashi et al. [4] concluded that the level of pentosidine in sera of RA patients reflects the activity of the disease, as the serum level of pentosidine correlated with clinical indices of RA activity, as well as with levels of serum markers of inflammation (CRP, ESR) and the level of rheumatoid factor (RF).

AGE are immunogenic; the preparation of polyclonal antibodies to pentosidine was announced by two groups within the past decade [13,14]. The first monoclonal antibody (mAb) to AGE, designated 6D12, was prepared in 1991 [15], but the corresponding antigen was identified only five years later as CML [1]. Later, the authors reported cross-reactivity of this mAb to N^ε^-(carboxyethyl)lysine (CEL) [16]. The development of the first specific mAb to CEL was reported in 2008 [17]. MAb to pentosidine is also commercially available (Cosmo Bio Co, Tokyo, Japan); however, we are not aware of any published information regarding its use in enzyme immunoassays.

We wished to investigate whether the reported increase of pentosidine levels in RA sera, and the positive correlation between pentosidine serum level and RA activity, also pertain to other AGE molecules. In contrast to HPLC, immunoassays are potentially more useful in routine laboratory practice. Thus, we decided to develop original immunoassays based on new mAbs to AGE.

## Methods

### Preparation of glycated antigens

Antigens were prepared according to the following three procedures.

Procedure A: reaction of protein carriers (BSA, bovine crystallin, porcine thyroglobulin, ovalbumin) with ribose. Proteins (50 mg/ml) were dissolved in 0.25 M phosphate buffer (pH 7.8) containing 0.5 M ribose. After addition of 2 drops of toluene, the samples were incubated for 6 weeks at 37°C. All samples were dialyzed extensively against phosphate-buffered saline (PBS) after 6 weeks to remove free sugar, then incubated again at 37°C for another 2 weeks to allow formed glycation products to ripen.

Procedure B: reaction of protein carriers with glyoxylic acid in the presence of sodium cyanoborohydride [18]. This reaction generates mainly CML.

Procedure C: reaction of protein carriers with pyruvate in the presence of sodium cyanoborohydride [19]. This reaction generates mainly CEL.

### Preparation and selection of monoclonal antibodies

**Figure 1:**
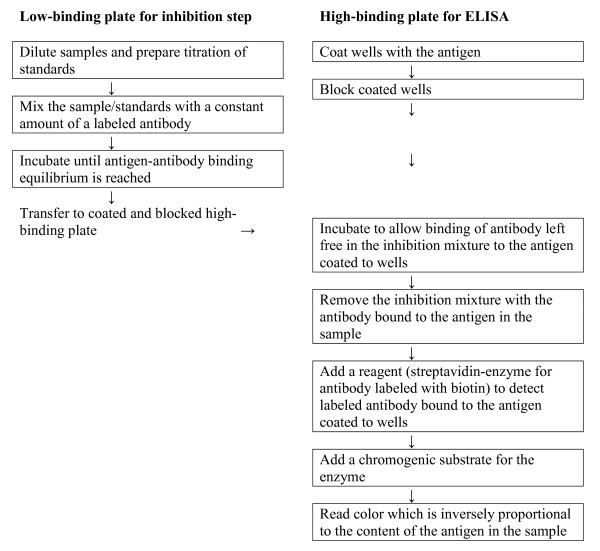
**Flow chart showing steps of the indirect inhibition ELISA**.

Splenocytes of Balb/c mice immunized with particular antigens were electrofused [20] with cells from the myeloma line Sp2/0. Hybridoma supernatants were screened over several subsequent rounds to gradually focus the selection. 1) Screening against the immunizing hapten, bound to a different protein carrier. 2) Screening against all non-modified protein carriers (BSA, crystallin, thyroglobulin, ovalbumin) and these same protein carriers modified by glycation ("ribosylation," change of lysine to CML or CEL), coated to a solid phase. 3) Selected pairs of glycated carriers were tested for the ability to mutually inhibit binding of corresponding antibodies to coated antigens. In this step, we compared the affinity of antibodies for antigens presented either in solid or liquid phase. The requirement of an antibody with similar affinity for antigen presented in solid or liquid phases is necessitated by its intended use in an inhibition enzyme-linked immuno-sorbent assay (ELISA) depicted in the flow chart shown in Figure 1. The interaction of an antigen with an antibody that occurs in an inhibition mixture established on a low-binding plate (left side of the flow chart) takes place in liquid phase; the interaction of the antigen with free antibody, that occurs after transferring the inhibition mixture onto a high-binding plate coated with the antigen (right side of the flow chart), takes place in solid phase. It is important that antigen-antibody complexes that formed in the liquid phase should remain stable during this latter step. 4) Concentrations of antigens used for coating plates and dilutions of culture media containing particular antibodies were optimized by titration. Selected conditions were used to test the inhibition of mAb binding to corresponding antigens coated to plates by diluted human sera. This test directly addressed the presence of measurable hapten levels in human serum (i.e., use of the corresponding mAb for analysis of human sera by inhibition ELISA).

Two mAbs were selected for further work: 103-E3 (unidentified antigen formed in the reaction of proteins with ribose), and 8-C1 (CEL). Both these mAbs are IgG_1_, kappa. These mAbs were isolated from culture media on Protein G, labeled with biotin, and used to develop inhibition ELISAs.

### Development of ELISAs and their characterization

We have developed an indirect inhibition ELISA for use with our mAbs. The steps for this procedure are shown in Figure 1.

We have used an AGE-modified high-molecular-weight protein as a temporal working standard, expressing results as its weight equivalents. Standards were either BSA glycated with ribose (Rib-BSA) for mAb 103-E3, or CEL-crystallin for mAb 8-C1. Either the same (Rib-BSA for mAb 103-E3) or the next from the set of proteins modified with corresponding AGE (CEL-ovalbumin for mAb 8-C1) was used as an antigen for coating high-binding plates. Maximum amplification of the signal was achieved by using a combination of biotin-labeled mAb and ultra-sensitive streptavidin-peroxidase polymer for detection.

It was necessary to use extreme dilution of antibodies in the inhibition mixture to achieve the required sensitivity in both assays, as the antigen concentrations in the samples (sera) are very low. Therefore, we blocked not only the coated MaxiSorp plates used for ELISA, but also the low-binding plates used for incubation of the inhibition mixture. We used synthetic polymer polyvinylpyrrolidone (PVP) [21] for blocking the low-binding plates, and we also added it to all solutions used for dilution of antibodies and standards. Results can be affected, as any protein added to the inhibition mixture can hypothetically bind the antibody due to its natural content of AGE. Coated MaxiSorp plates were blocked with BSA; at this stage, the results can no longer be affected by any possible natural content of AGE in albumin.

**Figure 2:**
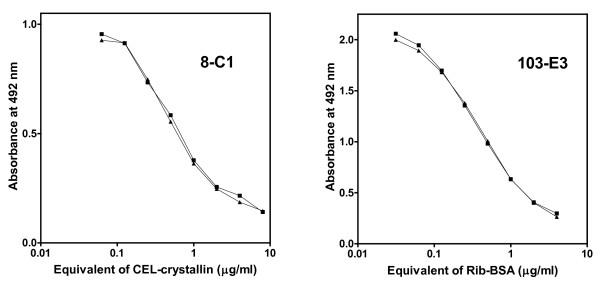
**Representative examples of calibration curves of inhibition ELISAs with mAbs 8-C1 and 103-E3**. Duplicate calibration curves were established on one plate.

Both ELISAs are very sensitive; titration curves of sera diluted 20×, 40×, and 80× in the inhibition mixture were practically parallel with the 4-parameter calibration curve that was obtained by titration of standards. Representative examples of both calibration curves are shown in Figure 2. Intra-assay variability of both ELISAs was approximately 5%. Inter-assay variability was higher (10-20%), possibly due to an operator change during the course of the study. Sera sets that should be directly compared were measured, if possible, on one plate, to avoid possible bias from high inter-assay variability. For larger sets that had to be accommodated on several plates, coded sera samples of all diagnoses were grouped, and then the order of all samples was randomized. Results measured on different plates were always normalized to the mean of the octuplicate control (pooled) serum that was included on every plate. Results were taken from one of at least two independent measurements, performed at least one week apart.

Labeled mAbs stored at 4°C, and standards divided into small aliquots and stored at -20°C are stable for a minimum of several months. Serum samples are stable for one week if stored unfrozen at 4°C; frozen serum samples (-70°C) are stable for a minimum of 10 freeze/thaw cycles. However, levels measured in the same sample differ depending on the storage method. Following the first freeze/thaw cycle, levels measured in frozen sera were approximately 10% higher than samples that were never frozen. This change of approximately 10% remained unaltered during further freeze/thaw cycles. This phenomenon can be explained, in our opinion, by partial denaturation of serum proteins, leading to increased epitope accessibility for the antibody.

Since pentosidine prevails among species generated during *in vitro *glycation of proteins with ribose [18], we measured the level of 103-E3 antigen in a collection of sera with a known concentration of pentosidine determined by HPLC [8]. However, we found no correlation between the concentration of 103-E3 antigen measured with ELISA and the concentration of pentosidine (Braun and Vilim, unpublished results). This observation indicates that ribose-derived 103-E3 antigen is different from pentosidine. Since pentosidine is a product of glycoxidation, this conclusion is consistent with our addition of a drop of toluene to the ribosylation mixture to avoid bacterial contamination. Thus, conditions were created that preferred simple glycation over glycoxidation by blocking oxygen access to the glycation mixture.

### Definitive protocol for inhibition ELISA with monoclonal antibody 103-E3

#### In-house prepared reagents

BSA modified with ribose (Rib-BSA); mAb 103-E3 labeled with biotin (EZ-LINK™ NHS-LC-biotin, 21336, Pierce, Rockford, IL, USA) to molar ratio of IgG:biotin approximately 1:50 (b-103-E3).

#### Plate coating

"High-binding" MaxiSorp plates (F96, 442404, Nunc A/S, Roskilde, Denmark) were coated by Rib-BSA, 5 μg/ml in 50 mM carbonate/bicarbonate buffer (pH 9.2), 100 μl/well. After addition of the antigen, the plate was covered with plate-sealing tape, incubated on an orbital shaker at room temperature (RT) for 2 hours, then stored at 4°C overnight.

#### Inhibition step (Day 1)

"Low-binding" plates for the inhibition step (either non-treated polypropylene U96 PP MicroWell Plates, 267385, Nunc, or non-treated polystyrene F96 MicroWell Plates, 269620, Nunc) were blocked by 1% PVP (PVP40, Sigma-Aldrich s.r.o., Praha, Czech Republic) in PBS (300 μl/well, 2 h RT on the orbital shaker). Blocked low-binding plates were immediately washed 3× with PBS/0.05% Tween 20 (TPBS) and used to set up an inhibition mixture. Standard (Rib-BSA) was diluted as well as titrated in PBS/1% PVP/0.02% sodium azide (PBS/PVP/azide); titration started at 8 μg/ml, and the resulting volume after titration was 70 μl/well. Sera were diluted 10× in PBS/PVP/azide and assayed in duplicate. 70 μl b-103-E3 diluted 100,000× in PBS/PVP/azide were added to each well containing 70 μl of standard or diluted serum. The resulting dilutions of reagents in the inhibition mixture were as follows: standard (Rib-BSA) titrated to concentrations of 4, 2, 1, 0.5, 0.25, 0.125, 0.0625, and 0.03125 μg/ml; sera diluted 20×; b-103-E3 diluted 200,000× (approximately 10 ng/ml). Final volume of the inhibition mixture was 140 μl/well. The low-binding plate with the inhibition mixture was covered with plate-sealing tape, incubated on the orbital shaker at RT for 2 hours, and stored at 4°C overnight.

#### ELISA (Day 2)

The coated MaxiSorp plate was blocked (5% BSA in PBS, 120 μl/well) for 2 h at RT on the orbital shaker, and washed 5× with TPBS. 100 μl of the inhibition mixture was transferred to the coated and blocked MaxiSorp plate and incubated on the orbital shaker at RT for 2 hours. The plate was washed 5× with TPBS, and 100 μl of Streptavidin-Peroxidase Polymer (S2438, Sigma-Aldrich), diluted 2000× in PBS/1% PVP, was placed into the wells for 30 min at RT on the orbital shaker. Another wash 5× with TPBS followed, and 100 μl of peroxidase substrate (0.05% o-phenylenedamine dihydrochloride + H_2_O_2_, in citrate/phosphate buffer pH 5.0) were placed into the wells. Color development was stopped by the addition of 1 M H_2_SO_4_, 100 μl/well. Absorbance was measured at 492 nm. Concentration of the antigen in the samples was calculated using the 4-parameter calibration curve and expressed as equivalents of Rib-BSA preparation.

### Inhibition ELISA with monoclonal antibody 8-C1

#### In-house prepared reagents

Ovalbumin, containing a subset of lysine residues modified to CEL, used as a plate coating antigen; crystallin, with a subset of lysine residues modified to CEL, used as a standard; mAb 8-C1, labeled with biotin (EZ-LINK™ NHS-LC-biotin, Pierce) to molar ratio of IgG:biotin approximately 1:50 (b-8-C1).

The assay was developed and performed according to the same protocol as ELISA with mAb 103-E3; these two methods differ only with respect to the in-house prepared reagents and their dilutions.

### Subjects

This study included 45 RA patients, 40 patients with SLE, 48 patients with OA of the knee, and 51 healthy volunteers (HC). Informed consent for the study was given by all subjects, and the study was approved by the local ethics committee. Basic demographic, clinical and laboratory data corresponding to date of sera collection are summarized for all cohorts in Table 1. Clinical data include Disease Activity Score 28 (DAS28) [22] for RA patients, Systemic Lupus Erythematosus Disease Activity Index (SLEDAI) [23] for SLE patients, and grading according to Kellgren and Lawrence (K/L grade) [24] for OA patients. All RA patients were treated with anti-TNF-α therapy (infliximab) in combination with methotrexate (MTX). Infliximab was administered by intravenous infusion at a dosage of 3 mg/kg every 8 weeks; MTX was administered at a constant dosage of 10-20 mg/week. In addition, 40/45 RA patients (89%) received a constant dosage of glucocorticoids (either methylprednisolone 4-10 mg/day or prednisolone 2.5-10 mg/day or every other day). Sera were collected during the first year of treatment with infliximab; prior to serum collection, each RA patient received at least 4 infliximab infusions. This RA patient cohort was chosen for the treatment with infliximab due to extremely high disease activity, and because they failed to respond to previous treatment. The cohort also included a high proportion of non-respondents to the infliximab therapy. 87% of RA patients were RF positive, and 69% were anti-CCP positive.

**Table 1:** Major demographic, clinical, and laboratory characteristics of analyzed cohorts.

	RA	SLE	OA	HC
N (males/females)	45 (12/33)	40 (4/36)^¶^	48 (13/25)	51 (18/33)

age (years)	54 ± 13**	41 ± 13***	66 ± 10*	44 ± 15

disease duration (years)	13 ± 7	8 ± 8	8 ± 7	

DAS28	4.7 ± 1.2			

SLEDAI		11.8 ± 6.7		

K/L grade (I/II/III/IV)			2.6 ± 0.8 (2/22/17/7)	

CRP (mg/l)	15.0 ± 19.2^†^	4.0 ± 3.8	4.0 ± 3.3	

ESR (mm/h)	28 ± 21			

RA - rheumatoid arthritis; SLE - systemic lupus erythematosus; OA - osteoarthritis; HC - healthy volunteers (controls). Continuous characteristics are expressed as mean ± SD. ^¶^The proportion males/females in cohort of SLE patients was significantly different from other three cohorts that did not significantly differ from each other. *The mean age of OA patients was significantly higher than the mean age of all three other cohorts; **the mean age of RA patients was significantly lower then the mean age of OA patients but significantly higher than the mean age of subjects in SLE and HC cohorts; *** the mean age of subjects in SLE and HC cohorts did not significantly differ. ^†^The mean level of CRP in sera of RA patients was significantly higher than the mean level of CRP in sera of patients with SLE and OA.

### Laboratory methods

Sera were stored at -70°C until used. Levels of the following markers were determined using commercially-available kits, according to the manufacturers' protocol: high-sensitive CRP (CRP Latex, Olympus-Diagnostic, Olympus Czech Group, Praha, Czech Republic), anti-cyclic citrullinated peptide antibodies (Anti-CCP ELISA, Euro-Diagnostica AB, Malmö, Sweden), and rheumatoid factor (Particle Agglutination Test Serodia-RA, Fujirebio Inc., Tokyo, Japan).

### Statistical analysis

Sera levels of AGE of analyzed cohorts were compared using nonparametric tests; two cohorts were compared by the Mann-Whitney test, and three cohorts were compared by the Kruskal-Wallis test, followed by mutual comparison of all groups by Dunn's post test. The value 1.0 was assigned to samples with the antigen level below the limit of ELISA detection, to allow their inclusion in statistical analyses with nonparametric tests. The relationship between two variables was estimated by Spearman's nonparametric correlation. The proportion of males in all four cohorts was evaluated by chi-square test. P values lower then 0.05 were considered to be significant. Statistical analyses were performed using GraphPad Prism version 4.00 for Windows (GraphPad Software, San Diego, CA, USA).

## Results

### AGE levels in RA sera

**Figure 3:**
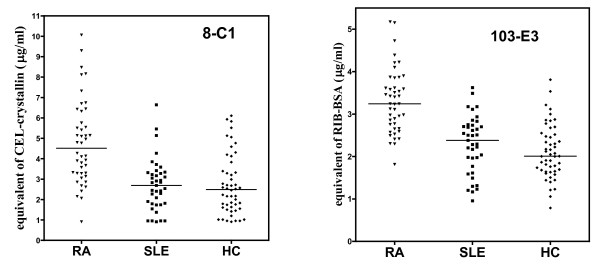
**AGE levels in sera of patients with rheumatoid arthritis and systemic lupus erythematosus, and controls**. The levels of N^ε^-(carboxyethyl)lysine (expressed as equivalents of CEL-crystallin) and 103-E3 antigen (expressed as equivalents of Rib-BSA) were measured in sera of patients with rheumatoid arthritis (RA) and systemic lupus erythematosus (SLE), and sera of healthy volunteers as controls (HC). The value 1.0 was assigned to samples with CEL levels of below the limit of ELISA detection with mAb 8-C1, to allow their inclusion in statistical analyses with nonparametric tests. Lines represent medians.

**Figure 4:**
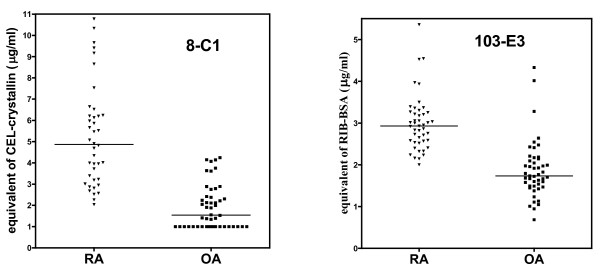
**AGE levels in sera of patients with rheumatoid arthritis and osteoarthritis**. The level of N^ε^-(carboxyethyl)lysine (expressed as equivalents of CEL-crystallin) and the level of 103-E3 antigen (expressed as equivalents of Rib-BSA) were measured in sera of patients with rheumatoid arthritis (RA) and osteoarthritis (OA). The value 1.0 was assigned to samples with CEL levels below the limit of detection of ELISA with mAb 8-C1, to allow their inclusion in statistical analyses with nonparametric test. Lines represent medians.

We observed accumulation of both measured glycation products (CEL and antigen binding to mAb 103-E3) in sera of RA patients (Figure 3). The difference was highly significant (P < 0.0001) when all three cohorts shown in Figure 3 (RA, SLE, HC) were simultaneously compared. The significant increase of AGE levels in RA sera versus SLE sera, as well as versus HC sera, was confirmed (P < 0.001) by Dunn's post-test. Levels of both antigens in SLE and HC sera did not differ. Means ± standard deviation (SD) determined for all three cohorts and both antigens are shown in Table 2. OA joined the list of diagnoses with significantly lower serum levels of antigenic AGE when compared with RA. OA sera were analyzed together with repeated analysis of RA sera, enabling direct comparison of both diagnoses (Figure 4); the difference was again highly significant (P < 0.0001). Means ± SD appear in Table 2.

**Table 2:** Levels of CEL and antigen binding to mAb 103-E3 determined in all investigated cohorts.

	RA *versus *SLE *versus *HC			RA *versus *OA	
	**RA**	**SLE**	**HC**	**RA**	**OA**

Equivalents of CEL-crystallin (μg/ml)	4.87 ± 1.989	2.92 ± 1.140	2.83 ± 1.437	5.413 ± 2.631	2.994 ± 2.023

Equivalents of Rib-BSA (μg/ml)	3.306 ± 0.755	2.287 ± 0.675	2.111 ± 0.620	2.993 ± 0.668	1.873 ± 0.692

RA - rheumatoid arthritis; SLE - systemic lupus erythematosus; OA - osteoarthritis; HC - healthy volunteers (controls). Results are expressed as means ± standard deviation of equivalents of CEL-crystallin or Rib-BSA. Samples with the level of measured antigen below the detection limit of the assay were not included in the calculation; these were 1 RA, 5 SLE, and 7 HC samples, respectively, assayed for CEL in the part "RA *versus *SLE *versus *HC", and 19 OA samples assayed for CEL and 1 OA sample assayed for 103-E3 antigen in the part "RA *versus *OA".

### Correlation of AGE levels in RA sera with clinical and laboratory data

Neither the levels of CEL nor the levels of 103-E3 antigen in RA sera correlated with RA activity measured by DAS28 score (Table 3). Levels of both antigens in RA sera did not correlate with age, gender, treatment with corticosteroids, levels of CRP, anti-CCP antibodies, or RF in sera (Table 3). The only significant correlation we observed (P = 0.0317) was the negative correlation between disease duration and concentration of 103-E3 antigen (Table 3). This correlation should not be considered significant, as we report the results of testing 7 independent null hypotheses for each ELISA; applying the Bonferroni correction for multiple hypothesis testing, a P-value accepted as significant at the 5% level should be less then 0.0073. However, also the P-value of the negative correlation between disease duration and CEL concentration was the lowest P-value calculated, although it did not reach statistical significance. Decrease of apparent AGE levels in RA sera with disease duration would make sense if, for example, autoantibodies reacting to AGE and interfering in assays gradually accumulated in RA sera during the course of the disease. Our preliminary data (Vilim and Vytášek, unpublished data) suggest that this hypothesis may be correct.

**Table 3:** Correlation between serum AGE levels and selected characteristics of patients with rheumatoid arthritis

	CEL		103-E3 antigen	
	**Spearman r**	**P**	**Spearman r**	**P**

DAS28	-0.0861	0.5783	-0.1032	0.5051

Age	-0.1367	0.3879	-0.0940	0.5485

Disease duration	-0.1945	0.2114	-0.3244	0.0317

CRP	-0.1204	0.4420	0.0971	0.5306

anti-CCP titer	-0.0974	0.5407	-0.1170	0.4604

RF titer	-0.1173	0.4651	0.0191	0.9043

corticosteroid dose	0.0472	0.7813	0.1884	0.2574

## Discussion

In the current study, we have extended the data on AGE in sera of RA patients. We determined AGE levels with ELISA, rather than the HPLC that has been used exclusively thus far. Additionally, we measured in sera two new AGE molecules, instead of only measuring pentosidine. To this purpose, we employed original in-house ELISAs with two new mAbs to AGE. We report the significant increase in mean levels of corresponding AGE in sera of RA patients.

Our results do not support the initially-published conclusion that AGE serum concentration correlates with RA activity and concentrations of inflammation markers. With the exception of disease duration, we did not identify associations of AGE levels with any RA attribute or inflammation marker included in this study. Indeed, upon examination of all published studies, correlation between serum/plasma pentosidine and laboratory markers/clinical indices appears equivocal. For example, four [6,9,10,12] of six studies confirmed statistically-significant positive correlations between serum pentosidine and CRP, but two [8,11] did not. Correlation between DAS28 and serum pentosidine was reproduced only in one study [9] of four [8-11].

In fact, this discrepancy among correlation analyses is not surprising. Perhaps a fluctuation in mean blood glucose (reducing sugars) levels within the time interval that precedes serum collection may be the reason. Glycated proteins in the blood are considered to provide an integrated measure of blood glucose over a scale of days to weeks, as they reflect the mean blood glucose level over a period of their half-life [25]. Since reactions that form AGE are not reversible, AGE slowly accumulate over the lifetime of a protein, and the extent of modification is thus a function of protein turnover. Presuming the random targeting of accessible lysines of serum proteins for glycation, the most modifications can be expected to be present on the most abundant serum protein, serum albumin. Indeed, the extent of albumin glycation accounts for more than half of total plasma protein glycation [26]; of the 59 lysines present in human serum albumin, 31 have been found to be glycated *in vivo *[27].

Specific albumin glycation depends on its half-life, and with an average life span of approximately 20 days, glycated albumin is considered to be an indicator of mid-term (2-3 weeks prior to serum sampling) glycemic control [28]. Hence, the correct approach for assessing correlation between serum AGE levels and RA activity would be to assess the correlation between a specific protein modification level and a synovitis, present in all joints in the body during the interval of this protein's half-life. Similarly, when calculating the correlation between serum AGE levels and laboratory inflammation markers, half-lives of a selected glycated protein and a selected inflammation marker in serum should be comparable, unless there is evidence that the level of the selected marker was stable over the given time interval. Accordingly, we also believe that a hypothetical age effect on the AGE levels we have measured is improbable. While the gradual accumulation of AGE with age on long-lived proteins is well-documented [29], in serum all proteins have short- to mid-term half-lives, and thus glycation would be affected by their actual fluctuations during this time interval, rather then by patient age. While the mean age of subjects in the RA cohort may be a cause of concern from the viewpoint of the effect of age, being higher than the mean age in the SLE and HC cohorts, the mean age of subjects in OA cohort was higher compared with RA cohort.

There is no doubt that AGE levels in RA sera/plasma are increased, as this increase has been reported for pentosidine in six papers by five independent groups. In fact, RA is the disease with the highest mean level of pentosidine in serum/plasma of all diseases investigated so far, even higher than the mean level found in sera/plasma of diabetic patients [5,12]. This last finding was corroborated for both AGE measured here (Vilim and Vytášek, unpublished results). All patients in our RA cohort were treated with MTX and with infliximab. Both of these treatments have been independently shown to reduce levels of pentosidine in RA sera [9,10], and thus the levels of AGE we have measured here would likely be even higher in untreated RA patients.

What is the participation of the receptor for advanced glycation end-products (RAGE)? AGE, *via *RAGE signaling, amplify the inflammatory response [30], and in this way likely play a role in sustaining chronic inflammation. It has to be borne in mind, however, that RAGE binds several discrete families of structurally dissimilar ligands in addition to AGE. Furthermore, the AGE designation also involves a diverse class of heterogeneous compounds, and not all AGE activate RAGE. More RAGE ligands likely exist within the AGE group, but only CML has been unambiguously demonstrated to be a RAGE ligand so far [31]. Pentosidine, on the other hand, does not seem to bind RAGE [18]. Although accumulation of AGE generally can contribute to the perpetuation of chronic inflammation in RA synovia, the cause of their accumulation remains obscure.

Oxidative stress resulting in the generation of free radicals in inflamed joints has been suggested to cause increased formation of AGE in RA. This hypothesis seems to be in agreement with our observation that the mean DAS28 value in our RA patient cohort at the time of sera collection still indicated increased disease activity. Additionally, the mean level of CRP in RA sera was also significantly increased compared with SLE and OA sera. However, CRP and AGE levels in RA sera did not correlate and seemed to be independent of each other (Table 3) while, as expected, CRP levels correlated significantly with DAS28 (data not shown).

An alternative hypothesis of autoimmunity, currently gaining experimental support, would also explain the available data. We propose to look for an opposite causality; rather than RA causing increased glycation, perhaps individuals prone to nonenzymatic glycation of proteins (for whatever reason) may be more likely to develop RA. This working hypothesis is plausible and attractive, since glycation forms new antigenic determinants on proteins. This type of connection has been already demonstrated for a different class of posttranslational protein modification, represented by conversion of arginine to citrulline, in a large subset of RA patients [32]. RA is an extremely complex disease, with an etiology involving the interplay of (at least) genetic predisposition, environmental factors, and immunity to self molecules made immunogenic by posttranslational modification [33]. Recent reviews contain many examples of different posttranslational modifications within target proteins leading to the breaking of immunological tolerance and induction of autoimmunity [34-37]. The process would ultimately lead to the development of "genuine" autoantibodies by epitope spreading.

It has long been known that glycosylation patterns of the IgG constant region are skewed toward less galactosylated variants in patients with RA [38]. It was hypothesized that incomplete galactosylation may be responsible for the induction of RF in RA [39] but the support for this hypothesis is weak and indirect [40,41]. However, RA sera contain autoantibodies against agalactosyl IgG [42,43]. Another group reported detection of autoantibodies to IgG that has been modified by binding AGE in sera of RA patients [44,45]. Hence, abnormal glycation of IgG in patients with RA was already related to induction of autoantibodies. We believe that the possibility this effect pertains also other serum proteins merits experimental testing. The antibodies we have developed could become useful tools in this effort.

## Conclusion

We report the highly specific increase of two advanced glycation end-products in sera of patients with rheumatoid arthritis. This increase could be explained neither by RA activity nor by inflammation. We propose a working hypothesis that presumes the existence of a link between AGE formation and the induction of autoimmunity.

## Abbreviations

AGE: advanced glycation end-products; CML: N^ε^-(carboxymethyl)lysine; CEL: N^ε^-(carboxyethyl)lysine; RA: rheumatoid arthritis; SLE: systemic lupus erythematosus; OA: osteoarthritis; HC: healthy control; mAb: monoclonal antibody; RF: rheumatoid factor; ELISA: enzyme-linked immuno-sorbent assay; HPLC: high-performance liquid chromatography; MTX: methotrexate; Rib-BSA: bovine serum albumin modified with ribose; b-103-E3: monoclonal antibody 103-E3 labeled with biotin; b-8-C1: monoclonal antibody 8-C1 labeled with biotin; PVP: polyvinylpyrrolidone; PBS: phosphate-buffered saline; TPBS: phosphate-buffered saline with 0.05% Tween 20; RAGE: receptor for advanced glycation end-products.

## Competing interests

The authors declare that they have no competing interests.

## Authors' contributions

RV developed monoclonal antibodies. LŠ assembled, characterized, and managed the cohort of patients with rheumatoid arthritis. VV designed and organized the study and drafted a manuscript. All authors read and approved the final manuscript.

## Pre-publication history

The pre-publication history for this paper can be accessed here:

http://www.biomedcentral.com/1471-2474/11/83/prepub

## References

[B1] Ikeda K Higashi T Sano H Jinnouchi Y Yoshida M Araki T Ueda S Horiuchi S N^ε^-(carboxymethyl)lysine protein adduct is a major immunological epitope in proteins modified with advanced glycation end products of the Maillard reaction Biochemistry 1996 35 8075 8083 10.1021/bi9530550 8672512

[B2] Sell DR Monnier VM Structure elucidation of a senescence cross-link from human extracellular matrix. Implication of pentoses in the aging process J Biol Chem 1989 264 21597 21602 2513322

[B3] Zhang Q Ames JM Smith RD Baynes JW Metz TO A perspective on the Maillard reaction and the analysis of protein glycation by mass spectrometry: probing the pathogenesis of chronic disease J Peoteome Res 2009 8 754 769 10.1021/pr800858h PMC2642649 19093874

[B4] Takahashi M Suzuki M Kushida K Miyamoto S Inoue T Relationship between pentosidine levels in serum and urine and activity in rheumatoid arthritis Brit J Rheumatol 1997 36 637 642 10.1093/rheumatology/36.6.637 9236672

[B5] Rodriguez-Garcia J Requena JR Rodriguez-Segade S Increased concentrations of serum pentosidine in rheumatoid arthritis Clin Chem 1998 44 250 255 9474020

[B6] Chen JR Takahashi M Suzuki M Kushida K Miyamoto S Inoue T Comparison of the concentration of pentosidine in the synovial fluid, serum and urine of patients with rheumatoid arthritis and osteoarthritis Rheumatology (Oxford) 1999 38 1275 1278 10.1093/rheumatology/38.12.1275 10587559

[B7] Hein GE Köhler M Oelzner P Stein G Franke S The advanced glycation end-product pentosidine correlates to IL-6 and other relevant inflammatory markers in rheumatoid arthritis Rheumatol Int 2005 26 137 141 10.1007/s00296-004-0518-1 15580352

[B8] Šenolt L Braun M Vencovský J Šedová L Pavelka K Advanced glycation end-product pentosidine is not a relevant marker of disease activity in patients with rheumatoid arthritis Physiol Res 2007 56 771 777 17298211 10.33549/physiolres.931147

[B9] Kageyama Y Takahasi M Nagafusa T Torikai E Nagano A Methotrexate reduces the levels of pentosidine and 8-hydroxy-deoxy guanosine in patients with rheumatoid arthritis Mod Rheumatol 2007 17 398 402 10.1007/s10165-007-0607-6 17929132

[B10] Kageyama Y Takahasi M Ichikawa T Torikai E Nagano A Reduction of oxidative stress marker levels by anti-TNFα antibody, infliximab, in patients with rheumatoid arthritis Clin Exp Rheumatol 2008 26 73 80 18328150

[B11] Kageyama Y Takahasi M Nagafusa T Torikai E Nagano A Etarnecept reduces the oxidative stress marker levels in patients with rheumatoid arthritis Rheumatol Int 2008 28 245 251 10.1007/s00296-007-0419-1 17661050

[B12] Miyata T Ishiguro N Yasuda Y Ito T Nangaku M Iwata H Kurokawa K Increased pentosidine, an advanced glycation end-product, in plasma and synovial fluid from patients with rheumatoid arthritis and its relation with inflammatory markers Biochem Biophys Res Commun 1998 244 45 49 10.1006/bbrc.1998.8203 9514872

[B13] Izuhara Y Miyata M Ueda Y Suzuki D Asahi K Inagi R Sakai H Kurokawa K A sensitive and specific ELISA for plasma pentosidine Nephrol Dial Transplant 1999 14 576 580 10.1093/ndt/14.3.576 10193802

[B14] Sanaka T Funaki T Tanaka T Hoshi S Niwayama J Taitoh T Nishimura H Higuchi C Plasma pentosidine levels measured by a newly developed method using ELISA in patients with chronic renal failure Nephron 2002 91 64 73 10.1159/000057606 12021521

[B15] Horiuchi S Araki N Morino Y Immunochemical approach to characterize advanced glycation end products of the Maillard reaction. Evidence for the presence of a common structure J Biol Chem 1991 266 7329 7332 2019568

[B16] Koito W Araki T Horiuchi S Nagai R Conventional antibody against N^ε^-(carboxymethyl)lysine (CML) shows cross-reaction to N^ε^-(carboxyethyl)lysine (CEL): immunochemical quantification of CML with a specific antibody J Biochem (Tokyo) 2004 136 831 837 10.1093/jb/mvh193 15671494

[B17] Nagai R Fujiwara Y Mera K Yamagata K Sakashita M Takeya M Immunochemical detection of N^ε^-(carboxyetlyl)lysine using a specific antibody J Immunol Met 2008 332 112 120 10.1016/j.jim.2007.12.020 18242632

[B18] Valencia JV Weldon SC Quinn D Kiers GH DeGroot J TeKoppele JM Hughes TE Advanced glycation end product ligands for the receptor for advanced glycation end products: biochemical characterization and formation kinetics Anal Biochem 2004 324 68 78 10.1016/j.ab.2003.09.013 14654047

[B19] Ahmed MU Brinkmann Frye E Degenhardt TP Thorpe SR Baynes JW N^ε^-(carboxyetlyl)lysine, a product of the chemical modification of proteins by methylglyoxal, increases with age in human lens proteins Biochem J 1997 324 565 570 9182719 10.1042/bj3240565 PMC1218467

[B20] Stenger DA Kubiniec RT Purucker WJ Liang H Hui SW Optimization of electrofusion parameters for efficient production of murine hybridomas Hybridoma 1988 7 505 518 10.1089/hyb.1988.7.505 3198135

[B21] Haycock JW Polyvinylpyrrolidone as a blocking agent in immunochemical studies Anal Biochem 1993 208 397 399 10.1006/abio.1993.1068 8095775

[B22] Prevoo ML van T'Hof MA Kuper HH van Leeuwen MA Putte LB van De van Riel PL Modified disease activity scores that include twenty-eight-joint counts. Development and validation in a prospective longitudinal study of patients with rheumatoid arthritis Arthritis Rheum 1995 38 44 48 10.1002/art.1780380107 7818570

[B23] Bombardier C Gladman DD Urowitz MB Caron D Chang CH Derivation of the SLEDAI. A disease activity index for lupus patients. The committee on prognosis studies in SLE Arthritis Rheum 1992 35 630 640 10.1002/art.1780350606 1599520

[B24] Kellgren JH Lawrence JS Radiological assesment of osteoarthritis Ann Rheum Dis 1957 16 494 501 10.1136/ard.16.4.494 13498604 PMC1006995

[B25] Misciagna G De Michele G Trevisan M Non enzymatic glycated proteins in the blood and cardiovascular disease Curr Pharm Des 2007 13 3688 3695 10.2174/138161207783018545 18220807

[B26] Baynes JW Thorpe SR Murtiashaw MH Nonenzymatic glucosylation of lysine residues in albumin Methods Enzymol 1984 106 88 98 full_text 6436646 10.1016/0076-6879(84)06010-9

[B27] Zhang Q Tang N Schepmoes AA Phillips LS Smith RD Metz TO Proteomic profiling of nonenzymatically glycated proteins in human plasma and erythrocyte membranes J Peoteome Res 2008 7 2025 2032 10.1021/pr700763r PMC2731429 18396901

[B28] Schleicher ED Olgemöller B Wiedenmann E Gerbitz KD Specific glycation of albumin depends on its half-life Clin Chem 1993 39 625 628 8472356

[B29] Verzijl N DeGroot J Thorpe SR Bank RA Shaw N Lyons TJ Bijlsma JWJ Lafeber FPJG Baynes JW TeKoppele JT Effect of collagen turnover on the accumulation of advanced glycation end products J Biol Chem 2000 275 39027 39031 10.1074/jbc.M006700200 10976109

[B30] Sparvero LJ Asafu-Adjei D Kang R Tang D Amin N Im J Rutledge R Lin B Amoscato AA Zeh HJ Lotze MT RAGE (receptor for advanced glycation endproducts), RAGE ligands, and their role in cancer and inflammation J Transl Med 2009 7 10.1186/1479-5876-7-17 19292913 PMC2666642

[B31] Kislinger T Fu C Huber B Qu W Taguchi A Yani SD Hofmann N Yan SF Pischetsrieder M Stern D Schmidt AM N^ε^-(carboxymethyl)lysine adducts of proteins are ligands for receptor for advanced glycation end products that activate cell signaling pathways and modulate gene expression J Biol Chem 1999 274 31740 31749 10.1074/jbc.274.44.31740 10531386

[B32] van Venrooij WJ Prujin GJM Citrullination: a small change for a protein with great consequences for rheumatoid arthritis Arthritis Res Ther 2000 2 249 251 10.1186/ar95 PMC130012 11094435

[B33] Klareskog L Stolt P Lundberg K Källberg H Bengtsson C Grunewald J Rönnelid J Erlandsson Harris H Ulfgren A-K Rantapää-Dahlquist S Eklund A Padyukov L Alfredsson L the Epidemiological Investigation of Rheumatoid Arthritis Study Group A new model for an etiology of rheumatoid arthritis. Smoking may trigger HLA-DR (shared epitope)-restricted immune reactions to autoantigens modified by citrullination Arthritis Rheum 2006 54 38 46 10.1002/art.21575 16385494

[B34] Doyle HA Mamula MJ Posttranslational protein modifications: new flavors in the menu of autoantigens Curr Opin Rheum 2002 14 244 249 10.1097/00002281-200205000-00009 11981321

[B35] Doyle HA Mamula MJ Posttranslational modifications of self-antigens Ann N Y Acad Sci 2005 1050 1 9 10.1196/annals.1313.001 16014515

[B36] Atassi MZ Casali P Molecular mechanisms of autoimmunity Autoimmunity 2008 41 123 132 10.1080/08916930801929021 18324481

[B37] Eggleton P Haigh R Winyard PG Consequence of neo-antigenicity of the "altered self" Rheumatology (Oxford) 2008 47 567 571 10.1093/rheumatology/ken014 18316337

[B38] Alavi A Axford JS Glyco-biomarkers: Potential determinants of cellular physiology and pathology Disease Markers 2008 25 193 205 10.1155/2008/863032 PMC3827810 19126964

[B39] Parekh RB Dwek RA Sutton BJ Fernandez DL Leung A Stanworth D Rademacher TW Mizoguchi T Taniguchi T Matsuta K Takeuchi F Nagano Y Miyamoto T Kobata A Association of rheumatoid arthritis and primary osteoarthritis with changes in the glycosylation pattern of total serum IgG Nature 1985 316 452 457 10.1038/316452a0 3927174

[B40] Tomana M Schrohenloher RE Koopman WJ Alarcon G Paul WA Abnormal glycosylation of serum IgG from patients with chronic inflammatory diseases Arthritis Rheum 1988 31 333 338 10.1002/art.1780310304 3358797

[B41] Čirič D Miloševič-Jovčič N Ilič V Petrovič S A longitudinal study of the relationship between galactosylation degree of IgG and rheumatoid factor titer and avidity during long-term immunization of rabbits with BSA Autoimmunity 2005 38 409 416 10.1080/08916930500241785 16278145

[B42] Das H Atsumi T Fukushima Y Shibuya H Ito K Yamada Y Amasaki Y Ichikava K Amengual O Koike T Diagnosic value of antiagalactosyl IgG antibodies in rheumatoid arthritis Clin Rheumatol 2004 23 218 222 10.1007/s10067-003-0860-9 15168148

[B43] Lu MC Hsieh SC Lai NS Li KJ Wu CH Yu CL Comparison of anti-agalactosyl IgG antibodies, rheumatoid factors, and anti-cyclic citrullinated peptide antibodies in the differential diagnosis of rheumatoid arthritis and its mimics Clin Exp Rheumatol 2007 25 716 721 18078619

[B44] Ligier S Fortin PR Newkirk MM A new antibody in rheumatoid arthritis targeting glycated IgG: IgM anti-IgG-AGE Br J Rheumatol 1998 37 1307 1314 10.1093/rheumatology/37.12.1307 9973155

[B45] Lucey MD Newkirk MM Neville C Lepage K Fortin PR Association between IgM response to IgG damaged by glyoxidation and disease activity in rheumatoid arthritis J Rheumatol 2000 27 319 323 10685791

